# TFEB ameliorates the impairment of the autophagy-lysosome pathway in neurons induced by doxorubicin

**DOI:** 10.18632/aging.101144

**Published:** 2016-12-16

**Authors:** Jose Felix Moruno Manchon, Ndidi-Ese Uzor, Shelli R. Kesler, Jeffrey S. Wefel, Debra M. Townley, Archana Sidalaghatta Nagaraja, Sunila Pradeep, Lingegowda S. Mangala, Anil K. Sood, Andrey S. Tsvetkov

**Affiliations:** ^1^ Department of Neurobiology and Anatomy, The University of Texas McGovern Medical School at Houston, Houston, TX 77030, USA; ^2^ The University of Texas Graduate School of Biomedical Sciences, Houston, TX 77030, USA; ^3^ Department of Neuro-Oncology, the University of Texas, MD Anderson Cancer Center, Houston, TX 77030, USA; ^4^ Department of Molecular and Cellular Biology, Baylor College of Medicine, Houston, TX 77030, USA; ^5^ Department of Gynecologic Oncology and Reproductive Medicine, the University of Texas, MD Anderson Cancer Center, Houston, TX 77030, USA; ^6^ Center for RNA Interference and Non-Coding RNA, the University of Texas, MD Anderson Cancer Center, Houston, TX 77030, USA; ^7^ Department of Cancer Biology, the University of Texas, MD Anderson Cancer Center, Houston, TX 77030, USA

**Keywords:** doxorubicin, chemotherapy, brain aging, autophagy, TFEB

## Abstract

Doxorubicin, a commonly used chemotherapy agent, induces severe cardio- and neurotoxicity. Molecular mechanisms of cardiotoxicity have been extensively studied, but mechanisms by which doxorubicin exhibits its neurotoxic properties remain unclear. Here, we show that doxorubicin impairs neuronal autophagy, leading to the accumulation of an autophagy substrate p62. Neurons treated with doxorubicin contained autophagosomes, damaged mitochondria, and lipid droplets. The brains from mice treated with pegylated liposomal doxorubicin exhibited autophagosomes, often with mitochondria, lipofuscin, and lipid droplets. Interestingly, lysosomes were less acidic in doxorubicin-treated neurons. Overexpression of the transcription factor EB (TFEB), which controls the autophagy-lysosome axis, increased survival of doxorubicin-treated neurons. 2-Hydroxypropyl-β-cyclodextrin (HPβCD), an activator of TFEB, also promoted neuronal survival, decreased the levels of p62, and lowered the pH in lysosomes. Taken together, substantial changes induced by doxorubicin contribute to neurotoxicity, cognitive disturbances in cancer patients and survivors, and accelerated brain aging. The TFEB pathway might be a new approach for mitigating damage of neuronal autophagy caused by doxorubicin.

## INTRODUCTION

Cognitive dysfunction often occurs in cancer patients during and after chemotherapy treatment. Chemotherapy may affect memory, attention, processing speed, and other cognitive functions [1]. Cognitive dysfunction sometimes persist for years, and cancer survivors experience a significant burden in coping with these impairments [2]. Many factors may contribute to cancer-related cognitive dysfunction [2, 3], but the direct neurotoxic effect of anti-neoplastic agents on the central nervous system is potentially the most important contributor. Commonly used anti-neoplastic agents or their metabolites directly interact with synaptic components, such as synaptic receptors and enzymes [4-10]. In addition, chemotherapy drugs, such as paclitaxel and vincristine, may damage neurons by reducing the health of mitochondria [11, 12]. Importantly, chemotherapy drugs may accelerate brain aging, thereby altering cognition and increasing the risk for neurodegenerative disorders [2, 13-16]. Mechanisms by which chemotherapy ages the brain are not clear.

Doxorubicin is an anti-cancer anthracycline compound that is used to treat several malignancies, including breast, esophageal and liver cancers, among others [17]. Anti-neoplastic properties of doxorubicin include interference with replication of DNA and RNA synthesis and the formation of free radicals, which leads to oxidative damage of cellular membranes. The drug has serious side effects, such as cardiomyopathy and brain damage. Cardiomyopathy is caused by oxidative stress, mitochondrial toxicity, and disturbances in proteostasis [17-20]. During chemotherapy, dexrazoxane, an iron chelator, can be used to protect the heart against the cardiotoxic effects of doxorubicin, although cardiotoxicity commonly limits dosages. Doxorubicin has restricted access to the brain, but still, it appears to penetrate the brain at levels sufficient to cause neurotoxicity, leading to pathological changes in the brain, such as significantly reduced brain connectivity and thinning of the cortex [2, 13-16, 21-24]. We recently demonstrated that doxorubicin damages DNA, synapses, and neurites in primary cultured neurons [25]. Despite the common clinical use of doxorubicin, the mechanisms by which doxorubicin exhibits its neuro-toxicity are not well studied. Importantly, neuro-protective drugs that would mitigate the brain damage are critically needed.

The bHLH-leucine zipper transcription factor EB (TFEB) regulates lysosomal biogenesis and autophagy. TFEB promotes autophagosomal–lysosomal fusion and prevents accumulation of autophagic organelles. TFEB activation is neuroprotective in models of neuro-degenerative disorders, such as Huntington's, Parkinson's, and Alzheimer's diseases [26-30]. If doxorubicin indeed damages the degradative systems in neurons, then upregulating TFEB might be neuroprotective.

In this study, we determined if doxorubicin induces the impairment of degradative pathways in cultured primary neurons. We discovered that autophagy is upregulated, but appears to be impaired and ineffective in clearing an autophagic marker, the p62 protein. With electron microscopy, we also discovered that vacuolar structures, autophagosomes, mitochondria, and lipid droplets accumulate in neurons treated with doxorubicin. In mice, pegylated liposomal doxorubicin (doxil) induced accumulation of autophagosomes and lipid droplets. Lysosomal pH is higher in doxorubicin-treated neurons. TFEB is neuroprotective for doxorubicin-treated neurons. Based on our findings, we conclude that treatment with doxorubicin leads to protein and organelle dyshomeostasis in neurons, which may contribute to cognitive impairments and accelerated brain aging induced by doxorubicin, and that targeting TFEB might be a therapeutic strategy.

## RESULTS

### Doxorubicin promotes formation of pre-autophagosomal complexes

Doxorubicin causes problems in the heart by altering proteostasis systems, such as autophagy, in cardio-myocytes [31]. In neurons, autophagy has a protective role, and it is dysregulated in many neurological disorders [32]. Some autophagic pathways, however, or overstimulated autophagy are associated with cytotoxicity, which indicates that autophagy in neurons can be helpful, harmful, or even both [33, 34]. We hypothesized that neurons upregulate autophagy in response to the treatment with doxorubicin. If so, we sought to determine if enhanced autophagy is a neuroprotective or neurotoxic mechanism.

**Figure 1:**
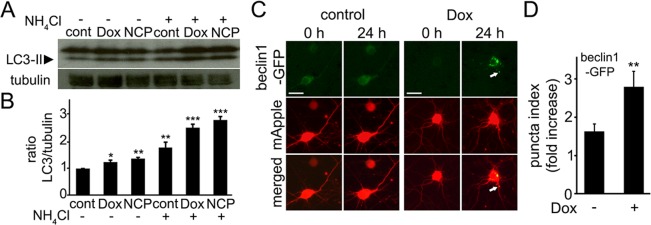
Doxorubicin induces autophagy in primary cortical neurons (**A**) Autophagy is induced in cultured primary cortical neurons by 50 nM doxorubicin (overnight) as reflected by the increased levels of LC3-II. Tubulin was used as a loading control. LC3-II accumulated in neurons treated overnight with 50 nM doxorubicin or 5 μM 10-NCP (an autophagy enhancer as positive control) with or without 10 mM NH_4_Cl, 4 h. LC3-II increased in doxorubicin-treated cells when NH_4_Cl was added reflecting enhanced autophagic flux. (**B**) Measurements of the LC3-II bands from (A). The LC3-II intensities were normalized to the tubulin loading control. *p<0.01, **p<0.001, ***p<0.0001 (ANOVA). Results were pooled from three independent experiments. (**C**) Doxorubicin promotes the formation of pre-autophagosomal complexes as reflected by beclin1-GFP-positive puncta. Cortical neurons were transfected with mApple (a morphology and viability marker) and beclin1-GFP, and treated with a vehicle or 50 nM doxorubicin (overnight). Note changes in beclin1-GFP localization, consistent with beclin1 relocalization to pre-autophagosomal structures. White arrow points a beclin1-GFP-positive structure in a neurite. Bar, 20 μm. (**D**) To score autophagy induction, the redistribution of beclin1-GFP into puncta, is reflected by the fold-increase of puncta index, which is the standard deviation among pixels within the cellular region of interest. The puncta index significantly increased in doxorubicin-treated neurons. **p<0.001 (t-test). Two hundred neurons were analyzed from two independent experiments.

Commonly used methods for monitoring autophagy are based on biochemical properties of an autophagy protein LC3. When an autophagosome forms, a proteolized form of LC3, LC3-I, is lipidated and can be detected as a mobility shift by western blotting. Increased LC3-II levels can also be a consequence of autophagic blockade at some step after the conversion of LC3-I to LC3-II. To determine if doxorubicin stimulates neuronal autophagy, we treated primary cortical neurons with doxorubicin or with doxorubicin and NH_4_Cl, which neutralizes the lysosomal pH and inhibits the last step of autophagy-mediated degradation, or with NH_4_Cl alone. LC3-II levels were higher in neurons incubated with the combination of doxorubicin and NH_4_Cl than in neurons incubated only with doxorubicin, indicating that autophagy is indeed upregulated (Fig. 1A,B).

Since LC3-I lipidation might reflect the induction of a pathway not directly related to autophagy [35], we decided to confirm that doxorubicin indeed promotes the formation of the pre-autophagosomal structures. Beclin1 is a part of the pre-autophagosomal complex and is often used as an autophagy marker [36, 37]. Beclin1-GFP and mApple, a marker of morphology, were expressed in two cohorts of primary cortical neurons. The first neuronal cohort was treated with a vehicle and the second cohort was treated with doxorubicin. As expected, doxorubicin induced the formation of beclin1-GFP-positive puncta (Fig. 1C,D), similar to what neuronal autophagy inducers do [37]. We recently showed that stimulating autophagy in neurons leads to the formation of beclin1-positive structures in the soma and neurites [37, 38]. In these experiments, we also observed beclin1-positive structures in the soma and neurites in doxorubicin-treated neurons.

### Levels of p62 increase in doxorubicin-treated neurons

**Figure 2:**
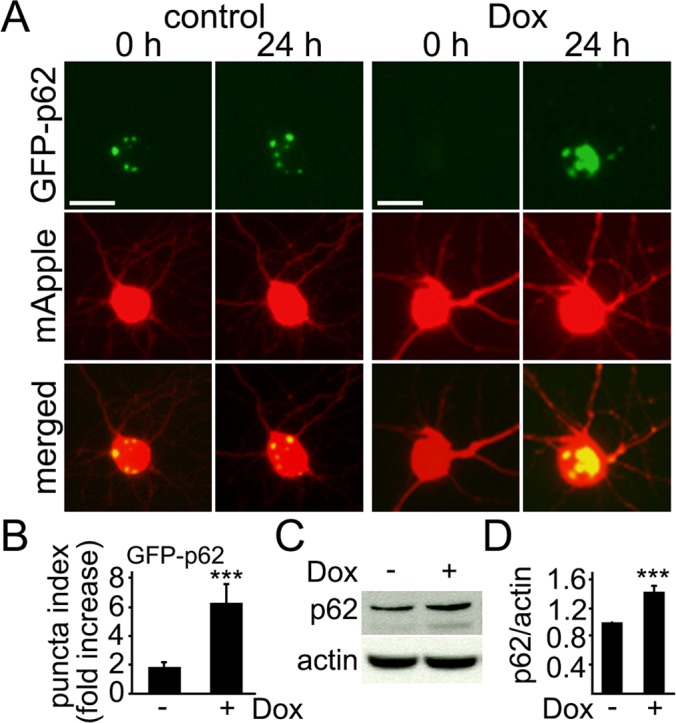
Levels of p62 increase in cultured cortical neurons treated with doxorubicin (**A**) Autophagy was impaired by doxorubicin. Cortical neurons were transfected with mApple (a morphology and viability marker) and GFP-p62. The first neuronal cohort was treated with a vehicle, and the second cohort was treated with 50 nM doxorubicin (overnight). Neurons were imaged before and after the treatments. Small aggresomes are sometimes formed in neurons. Note large inclusion bodies formed by GFP-p62 in doxorubicin-treated neurons. Bar, 10 μm. (**B**) Quantification of fluorescent images from (**A**). The fold-increase of the puncta index in neurons, which express GFP-p62, treated with a vehicle or with 50 nM doxorubicin (overnight). *** p<0.0001 (t-test). Two hundred neurons were analyzed from two independent experiments. (**C**) Endogenous p62 accumulated in cultured cortical neurons treated with 50 nM doxorubicin (overnight). Actin was used as a loading control. (**D**) Quantification of western blots from (**C**). The levels of p62 were normalized to actin. *** p<0.0001 (t-test). Results were pooled from four independent experiments.

The protein p62 (SQSTM1), which binds ubiquitin and LC3, is a selective substrate for autophagy [39, 40]. When autophagy is enhanced, the levels of p62 are reduced; impeding autophagy is associated with higher p62 levels. In cardiomyocytes, doxorubicin leads to accumulation of p62, indicating that autophagy is inhibited [31]. We, therefore, tested if doxorubicin affects the degradation of p62 in primary neurons. p62-GFP and mApple were expressed in two cohorts of primary cortical neurons; the first neuronal cohort was treated with a vehicle, and the second was treated with doxorubicin. Transfected neurons were followed longitudinally with an automated microscope. Interestingly, neurons sometimes formed p62-GFP-positive aggresomes under basal conditions, but doxorubicin induced the formation of very large inclusion bodies formed by p62-GFP, suggesting that p62 accumulates and autophagy is ineffective (Fig. 2A,B).

Next, we tested if the levels of endogenous p62 change in neurons treated with doxorubicin. Cultured cortical neurons were treated with either a vehicle or doxorubicin, lysed and analyzed for p62 (Fig. 2C,D). As expected, p62 levels were higher in neurons exposed to doxorubicin. Our data suggest that doxorubicin-treated neurons exhibit upregulated autophagy and there is some flux though autophagy, but autophagy is impaired and inefficient.

### Doxorubicin induces accumulation of vacuolar structures and organelles in neurons

**Figure 3:**
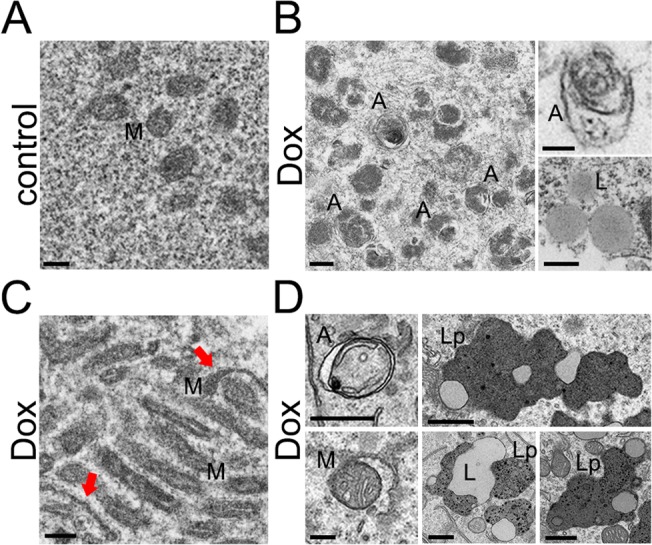
Accumulation of autophagosomes and organelles in primary cultured neurons and mouse brains induced by doxorubicin and doxil, respectively (**A**) An example of an electron micrograph of cultured cortical neurons treated with a vehicle (overnight). M, mitochondria. Bar, 200 nm. (**B**) Electron micrographs of cultured cortical neurons treated with 50 nM doxorubicin (overnight). Left panel: A, autophagosomes. Bar, 200 nm. Right upper panel: A, an autophagosome. Bar, 100 nm. Right lower panel: L, lipid droplets. Bar, 500 nm. (**C**) An example of an electron micrograph of cultured cortical neurons treated with 50 nM doxorubicin (overnight). Note an abnormally large cluster of mitochondria. Arrows note atypical mitochondria. Bar, 200 nm. (**D**) Electron micrographs of mouse brain exposed to doxil. Left panels: A, autophagosomes. M, a mitochondrion being engulfed by an autophagosome. Bar, 200 nm. Right panels: L, lipid droplets; Lp, lipofuscin. Bar, 500 nm.

Next, we examined whether the changes observed with the p62 reporter could be confirmed by electron microscopy. The first neuronal cohort was treated with a vehicle, and the second was treated with doxorubicin. Under control conditions, we found no autophagosomal structures (Fig. 3A); we have previously demonstrated the same result in untreated neurons [38]. In contrast, neurons treated with doxorubicin contained massive accumulations of vacuoles with often poorly identified content (Fig. 3B). Neurons also contained auto-phagosomes (Fig. 3B). Interestingly, we observed lipid droplets, which we never detected in control cells (Fig. 3B). Unexpectedly, more mitochondria were present in doxorubicin-treated cells (Fig. 3C). Some mitochondria were abnormally shaped or rounded (Fig. 3C). These data directly prove that doxorubicin impairs the de-gradative autophagic pathway, leading to accumulation of damaged organelles, autophagosomes, vacuoles, and lipid droplets.

### Doxil induces accumulation of autophagosomes and lipid droplets in mouse brains

We were impressed by the changes we observed in doxorubicin-treated cultured neurons, and wondered if we could detect these pathologies *in vivo*. Mice underwent a course of chemotherapy with doxil, the liposomal doxorubicin frequently used in human patients. Mice were then perfused, and their brains were analyzed by electron microscopy. Remarkably, we observed autophagosomes, which often contained mitochondria. Some autophagosomes were empty, as in neurodegenerative Huntington's disease [41] (Fig. 3D). We also detected the accumulation of lipid droplets and lipofuscin, a mixture of oxidized proteins and lipids, found in aged brains and in Batten disease (Fig. 3D) [42, 43]. These data directly confirm that the treatment with doxorubicin leads to the accumulation of damaged organelles, autophagosomes, and lipid droplets.

### Doxorubicin damages lysosomes in primary neurons

**Figure 4:**
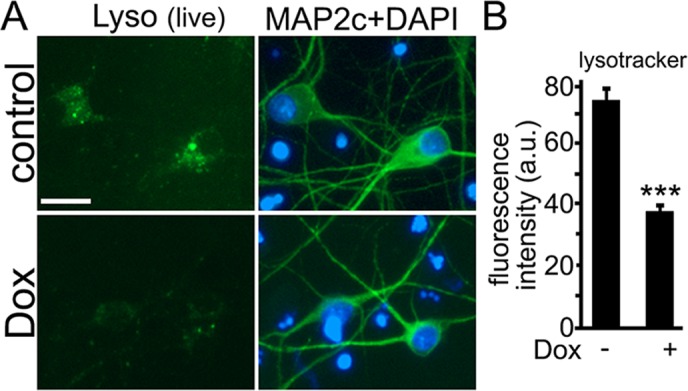
Doxorubicin raises the pH in lysosomes in cultured cortical neurons (**A**) Primary cortical neurons were treated with a vehicle or 50 nM doxorubicin (overnight), and stained with a green lysotracker dye, which stains acidic organelles in live cells. Cultures were imaged, fixed, and stained with an antibody for MAP2c and with the nuclear dye, and imaged again to confirm that observed lysosomes were located in neurons. Bar, 20 μm. (**B**) Quantification of fluorescent images with the green lysotracker dye from (**A**). The green signal was less in cultures treated with doxorubicin than in controls. *** p<0.0001 (t-test). Three hundred neurons were analyzed. Results were pooled from three independent experiments.

Doxorubicin is membrane-permeable in its basic form but impermeable when the molecule is protonated. Once inside the cell, doxorubicin enters acidic organelles such as lysosomes, in which doxorubicin is protonated and retained, leading to alkalinization of these organelles [44]. We hypothesized that doxorubicin impairs autophagy by affecting health of lysosomes in neurons. To test that, primary cortical neurons were treated with doxorubicin and stained with a green lysotracker dye, which stains acidic compartments in live cells. Remarkably, the green signal was significantly lower in cultures treated with doxorubicin than in controls (Fig. 4A, B). Cultures were then fixed and stained with MAP2c to confirm that visualized lysosomes belonged to neurons, and imaged again (Fig. 4A, B). Our data suggest that the drug affects lysosomal health in neurons, possibly by having an alkalinizing effect on the lysosomes, as it has in non-neuronal cells.

### TFEB overexpression increases survival of doxorubicin-treated neurons and mitigates damage of the autophagy-lysosome pathway

We previously discovered that the survival of individual neurons can be predicted by the efficiency of their proteostasis system and that enhancing proteostasis capacity with Nrf2, a transcription factor that regulates expression of stress response genes, promotes neuronal longevity [45]. Therefore, we hypothesized that normalizing degradative machinery in neurons treated with doxorubicin would also be neuroprotective. Since Nrf2, an obvious choice, also strongly upregulates a number of anti-oxidant genes that are cytoprotective for cancer cells [46], we searched for another neuro-protective candidate.

TFEB, the bHLH-leucine zipper transcription factor EB, regulates lysosomal biogenesis and autophagy. Several studies showed potential involvement of TFEB in cancer, although that appears to be related to the cancer type. In non–small cell lung carcinomas, TFEB expression is associated with poor cancer prognosis, although the mechanism is not clear [47]. Conversely, GDC-0941, an inhibitor of phosphatidylinositol-3-kinase, upregulates TFEB, leading to death of glio-blastoma cells [48].

**Figure 5:**
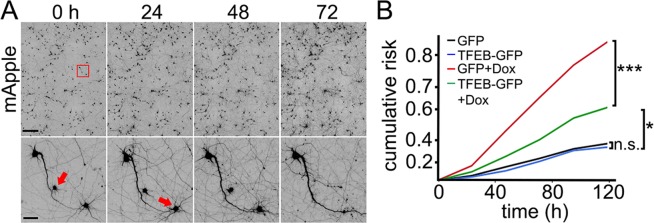
TFEB increases survival of doxorubicin-treated neurons (**A**) An example of longitudinal imaging and survival analysis. Primary cortical neurons transfected with mApple were followed with an automated microscope. Images collected every 24 h demonstrate the ability to return to the same field of neurons and track them over time. Each image is a montage of images captured in the center of one well of a 24- or 96-well plate. Scale bar is 400 μm. In the lower panel, images are zoomed in. Arrows represent two neurons that degenerate over time. Scale bar is 50 μm. (**B**) Two cohorts of primary cortical neurons transfected with mApple + GFP were treated either with a vehicle or 10 nM doxorubicin. Two cohorts transfected with mApple + TFEB-GFP were treated either with a vehicle or 10 nM doxorubicin. 16 hours after treatment, the four cohorts of neurons were imaged and tracked over 5 days. Risk of death associated with doxorubicin was calculated with JMP software. *p<0.01, ***p < 0.0001 (Log-Rank test), n.s., non-significant. One hundred fifty neurons were analyzed per condition. Results were pooled from two independent experiments.

We examined the survival of neurons that were treated with doxorubicin when TFEB was overexpressed. Four neuronal cohorts were transfected with mApple (a morphology and viability marker). The first and second neuronal cohorts were co-transfected with GFP; the third and fourth cohorts were co-transfected with TFEB-GFP. The first and the third cohorts were treated with a vehicle, and the second and the fourth were treated with doxorubicin. Neurons were followed longitudinally by automated microscopy, and the cumulative risk of neuronal death was calculated with longitudinal analysis (Fig. 5A,B). This technique allows us to track large cohorts of individual neurons over their lifetimes and to measure their survival with the statistical approaches used in clinical medicine [25, 37, 45, 49]. By tracking neurons over their lifetimes, we can determine whether applied drugs or expressed proteins contribute positively, negatively or neutrally to neuronal fate. We found that the expression of TFEB promotes neuronal survival of doxorubicin-treated neurons, as expected (Fig. 5B).

### 2-Hydroxypropyl-β-cyclodextrin promotes survival of doxorubicin-treated neurons and mitigates neuronal damage

**Figure 6:**
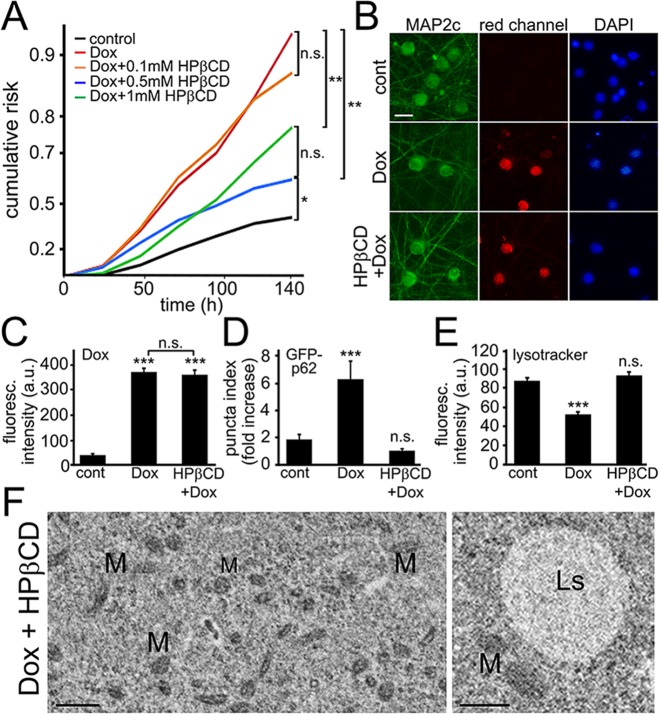
Cyclodextrin reduces neuronal damage induced by exposure to doxorubicin (**A**) Survival analysis of five neuronal cohorts transfected with mApple (a morphology and viability marker) were treated with a vehicle or with doxorubicin alone or in combination with HPβCD. The first neuronal cohort was treated with a vehicle. The second neuronal cohort was treated with 10 nM doxorubicin. The remaining three neuronal cohorts were treated with 10 nM doxorubicin in combination with 0.1 mM HPβCD (third neuronal cohort), 10 nM doxorubicin and 0.5 mM HPβCD (fourth neuronal cohort), and 10 nM doxorubicin and 1 mM HPβCD (fifth neuronal cohort). Neurons were tracked and imaged over 6 days. Risk of death associated with a treatment for each cohort was calculated with JMP software. *p<0.01, **p < 0.001 (Log-Rank test), n.s., non-significant. One hundred fifty neurons were analyzed per condition. Results were pooled from two independent experiments. (**B**) HPβCD does not prevent doxorubicin from binding to DNA. Cortical neurons were treated with 50 nM doxorubicin or with 50 nM doxorubicin and 0.5 mM HPβCD overnight, fixed, and stained for MAP2c (green) and with the nuclear Hoechst dye (blue), and imaged. Scale bar is 20 μm. (**C**) Images of fixed neurons from (**B**) were analyzed. ***p<0.0001 (t-test), n.s., non-significant. Three hundred neurons were analyzed per condition. Results were pooled from two independent experiments. (**D**) Three cohorts of cortical neurons were transfected with mApple and GFP-p62. The first neuronal cohort was treated overnight with a vehicle, the second cohort was treated with 50 nM doxorubicin (overnight), and the third cohort was co-treated with doxorubicin and 0.5 mM HPβCD (overnight). Quantification of fluorescent images revealed that HPβCD lowers the levels of GFP-p62 in doxorubicin-treated neurons. ***p < 0.0001 (t-test), n.s., non-significant. Two hundred neurons were analyzed. Results were from three independent experiments. (**E**) Cortical neurons were treated overnight with a vehicle or 50 nM doxorubicin or with a combination of doxorubicin and 0.5 mM HPβCD, and stained with a green lysotracker dye. HPβCD lowers the pH in in doxorubicin-treated neurons. ***p < 0.0001 (t-test), n.s., non-significant. Two hundred neurons were analyzed. Results were pooled from three independent experiments. (**F**) Electron micrographs of cultured cortical neurons co-treated with doxorubicin and 0.5 mM HPβCD (overnight). Left panel: Note that the cytoplasm lack the damage observed in neurons treated with doxorubicin alone. M, mitochondria. Bar, 1 μm. Right panel: Ls, lysosome; M, mitochondria. Bar, 500 nm.

2-Hydroxypropyl-β-cyclodextrin (HPβCD) has been actively investigated as a treatment for Niemann-Pick type C disease, a cholesterol storage disorder characterized by the accumulation of cholesterol and glycolipids in lysosomes. HPβCD exerts its protective functions, at least in part, by activating TFEB and, therefore, enhancing the function of the lysosomes and autophagic capacity [50]. We hypothesized that HPβCD mitigates autophagic damage in neurons associated with doxorubicin and improves their survival. In these experiments, neurons were transfected with mApple and treated with doxorubicin alone or co-treated with doxorubicin and HPβCD. Interestingly, HPβCD improved neuronal survival affected by doxorubicin, suggesting that HPβCD might be a co-therapy for treating cancer patients to reduce neuronal damage associated with the drug (Fig. 6A).

HPβCD, a cyclic molecule, may provide a hydrophobic environment and form a complex with doxorubicin, lowering its effective concentration. To determine if doxorubicin is still present in the culture media or, at least, if a potential complex of HPβCD/doxorubicin is weak, we investigated if HPβCD would prevent doxorubicin from binding to DNA. Indeed, we recently demonstrated that doxorubicin binds to nuclear DNA in cultured neurons [25]. Doxorubicin's spectrum has an emission peak at 595 nm and a shoulder at 650 nm, and can be visualized with a regular red fluorescent protein (RFP) filter. Neurons were incubated with HPβCD/doxorubicin or with doxorubicin alone, fixed, and the red nuclear staining was analyzed (Fig. 6B,C). Expectedly, HPβCD did not affect doxorubicin's binding to DNA.

Next, to confirm that HPβCD contributes to autophagy and is neuroprotective, we tested if the degradation of p62 is changed in the presence of HPβCD. Three neuronal cohorts were transfected with p62-GFP and mApple. The first cohort was treated with a vehicle; the second neuronal cohort was treated with doxorubicin; a third cohort was treated with doxorubicin and HPβCD. Indeed, co-treatment of doxorubicin and HPβCD lowered the levels of p62 in doxorubicin-treated neurons, suggesting that TFEB indeed enhanced the autophagic capacity in neurons (Fig. 6D).

Next, we investigated if the health of lysosomes can be improved by HPβCD. Neurons were pre-treated with vehicle or with doxorubicin or with a combination of doxorubicin and HPβCD and then stained with a green lysotracker dye. As expected, TFEB increased the intensity of the lysotracker dye in neurons, revealing that TFEB indeed improves the health of lysosomes in neurons treated with doxorubicin (Fig. 6E).

Would HPβCD improve the pathology that we observed by electron microscopy? Cultured neurons were pre-treated with HPβCD for several hours, and doxorubicin was added. Neurons were fixed and analyzed. Remarkably, we detected less pathology than in neurons treated with doxorubicin alone. The cytoplasm contained significantly fewer damaged organelles. No lipid droplets were detected (Fig. 6F). The neurons contained organelles that appeared to be young developing lysosomes. Given that HPβCD is in a clinical trial as a treatment for Niemann-Pick type C disease, our discovery was not unexpected, and is a promising candidate for rapid translation into the clinical setting. Would HPβCD improve the pathology and behavioral and cognitive impairments induced by doxorubicin? We plan to address these issues in a future manuscript.

## DISCUSSION

In this study, we showed that a commonly used chemotherapy drug, doxorubicin, impairs the autophagy-lysosome system in neurons. Affected neurons upregulated autophagy, perhaps in response to impaired organelles such as mitochondria, but failed to remove the damaged material. EM studies revealed that doxorubicin-treated neurons contain large amounts of vacuolar structures, autophagosomes, rounded mitochondria, and lipid droplets. Mice treated with doxil, the liposomal doxorubicin used in humans, also contained autophagosomes, lipid droplets, and lipo-fuscin-like material in their brains. The autophagy substrate p62 accumulates in these neurons. Lysosomal pH is higher in neurons exposed to doxorubicin than in vehicle-treated neurons. TFEB, the bHLH-leucine zipper transcription factor EB regulates lysosomal biogenesis, homeostasis, and fusion of autophagosomes and lysosomes. It also mitigates the damage associated with doxorubicin, and promotes neuronal survival. HPβCD, a molecule from the cyclodextrin compound class that upregulates TFEB, promotes health in neurons damaged by doxorubicin. Therefore, the TFEB pathway may be a therapeutic target for mitigating or preventing brain damage induced by doxorubicin.

Brain aging is a complex phenomenon characterized by the weakening of connectivity between the brain regions and reduced processing of information. Neurons lose their synapses, processes, and sometimes die. At the molecular level, there are multiple events, including DNA damage, accumulation of impaired organelles, oxidized lipids, lipofuscin, and protein aggregates [42]. Autophagy, one of the major proteostasis systems and the major organellostasis pathway, naturally declines with age, making neurons less capable of removing damaged or senescent organelles and abnormal proteins. This happens in successful or normative brain aging, and occurs at a significantly larger scale in the non-successful or pathogenic brain aging associated with neurodegenerative disorders. When autophagy is dysfunctional, lipid droplets accumulate in liver and other cell types [51, 52]. The exposure to certain chemicals, such as organophosphates, can accelerate brain aging [53]. Mitochondrial dysfunction also has a significant role in neurodegeneration and aging [54]. Clinical studies suggest that doxorubicin promotes brain aging. Cancer patients and survivors treated with doxorubicin exhibit weaker brain networks and more cognitive impairment than patients treated with other chemotherapy regimens [2, 13-16]. Furthermore, these brain networks are less resilient to aging and neurodegeneration after doxorubicin treatment. Patients treated with doxorubicin had acutely and chronically elevated molecular markers of senescence, including p16INK4a and ARF [55]. Previously, we demonstrated that doxorubicin induces DNA damage in neurons [25]. Here, we expanded on that work by showing specifically that the autophagy-lysosomal pathway is impaired in neurons treated with doxorubicin. Importantly, mouse brains exposed to doxil also showed autophagy-lysosomal pathology and the accumulation of lipofuscin. Residual lipofuscin can be found in the mouse brains as early as 12 months of mouse age, but is more evident when mice are aged [56, 57]. Therefore, since we used 4–6-month-old mice, doxil accelerated brain aging.

A rational combination of chemotherapeutic agents is the key to inducing death specifically in cancer cells, while sparing normal cells [58]. For example, a mix of metformin, an anti-diabetic drug, and rapamycin, an inhibitor of mTOR, is toxic for cancer cells but cytoprotective for non-cancerous cell types [59]. But would a therapeutic added to a chemotherapy cocktail protect normal cells also protect cancer cells? In addition to neurons, would cyclodextrins protect cancer cells from doxorubicin as well? Cyclodextrins are cyclic molecules that have been actively investigated for their properties to solubilize aromatic compounds. They provide a hydrophobic environment, encapsulate, and, therefore, solubilize hydrophobic molecules. As a result, cyclodextrins can be used as a delivery mechanism for poorly soluble drugs. Remarkably, methyl-β-cyclodextrin complexed with doxorubicin has been used in a mouse cancer model. Tumor growth was slowed, and survival increased in mice treated with the methyl-β-cyclodextrin/doxorubicin complex, compared to mice treated with either drug alone [60]. This indicates that methyl-β-cyclodextrin does not protect cancer cells from doxorubicin.

Cyclodextrins themselves have therapeutic potential: HPβCD is in a clinical trial to evaluate its potential for treating cholesterol storage disorders, in which lysosome function is abnormal. In a mouse model of lysosomal Niemann-Pick type C disease, HPβCD improves liver function, decreases neurodegeneration, and extends mouse lifespan [61]. HPβCD activates the TFEB transcription factor, the master regulator of the autophagy-lysosome pathway, thereby enhancing cellular degradative capacities [50].

In our study, we demonstrated that doxorubicin damages lysosomes and impairs autophagy in primary cultured neurons, and that HPβCD mitigates the damage. Our results identify a novel pathway of doxorubicin-mediated neurotoxicity and provide a potential therapy for mitigating or preventing brain damage in cancer patients treated with doxorubicin. Future studies in rodents will demonstrate if HPβCD improves brain pathology, and normalizes behavior and cognition impaired by doxorubicin.

## MATERIALS AND METHODS

### Chemicals and plasmids

Doxorubicin was from Selleckchem (Houston, TX). HPβCD was from Cayman Chemical Company (Ann Arbor, MI). 10-(4′-(N-diethylamino)butyl)-2-chloro-phenoxazine hydrochloride (10-NCP) was from Calbiochem (San Diego, CA). NH4Cl was from Biomatik (Wilmington, DE). Lysotracker Green DND-26 was from Life Technologies (Carlsbad, CA). Antibodies against actin (#3700, 1:1.000) and p62 (#5114, 1:1.000) were from Cell Signaling (Danvers, MA). Antibody against tubulin (#ab7751, 1:1.000) was from Abcam (Cambridge, MA). Antibody against MAP2c (#sc-74421, 1:100) was from Santa Cruz Biotechnologies (Dallas, TX), and antibody against LC3 (#PD014, 1:1.000) was from MBL (Woburn, MA). Anti-mouse Alexa Fluor 488-labeled (#A11001, 1:200) was from Life Technologies. Hoechst dye was from Santa Cruz Biotechnology. pEGFP-N1-TFEB was from Addgene (#38119, deposited by Dr. Shawn Ferguson, Yale School of Medicine). pGW1-beclin1-GFP was described [37]. pGW1-GFP-p62 was cloned from pMXs-puro GFP-p62, which was from Addgene (#38277, deposited by Dr. Noboru Mizushima, the University of Tokyo).

### Cell cultures

Cortices from rat embryos (E19) were dissected, digested with papain (Worthington Biomedical, Lakewood, NJ), resuspended in Optimem (Life Technologies) with 20 mM glucose (Biomatik, Wilmington, DE), and plated on 24-well tissue-culture plates (600.000 cells/well) coated with poly-D-lysine (EMD Millipore, Billeria, MA) as described [38, 49]. Neurons were maintained in Neurobasal Medium (Life Technologies), supplemented with B-27 (Life Technologies), GlutaMAX (Thermo Scientific, Waltham, CA), and penicillin-streptomycin (Sigma, St. Louis, MO).

### Immunocytochemistry

Cultured neurons were fixed with 4% paraformaldehyde (Santa Cruz Biotechnologies) for 15 min at room temperature. Cells were washed with PBS and permeabilized with 0.1% Triton X-100 (Santa Cruz Biotechnologies) in PBS for 10 min. Cells were washed before blocking overnight in PBS with 10% serum from the host species of a secondary antibody. Neurons were incubated overnight with antibodies against MAP2c (1:100) diluted in blocking buffer at 4°C. Cells were washed three times and incubated with anti-mouse-Alexa488 (Life Technologies) diluted in blocking buffer for 1 h at room temperature. Nuclei were stained with the Hoechst dye in PBS.

### Fluorescence microscopy and image analysis

Fixed neurons were imaged in automated fashion with the EVOS microscopy system (Life Technologies). The plate was placed on the EVOS microscope stage. The microscope automatically positions the 20x objective to the center of the first well of the 24-well tissue plate and collects fluorescence images with the RFP filter (mApple), the GFP filter (Beclin1; p62; lyso-tracker; TFEB), and the DAPI filter (Hoechst). Thereafter, the microscope moves the stage to each adjacent field in the well until a representative image is obtained from each well.

Puncta indexes reflect the redistribution of standard deviation of the fluorescence intensities of beclin1-GFP and GFP-p62 within soma and neurites before and after treatment [38]. Low puncta index represents diffuse localization, and high puncta index corresponds to punctate localization.

Neurons treated with vehicle or Dox were incubated with 400 nM Lysotracker Green for 10 min, imaged, and the green fluorescence intensity was quantified. Then cells were fixed and immunostained against MAP2c and stained with DAPI dye, as described above, to visualize morphology and nuclei of neurons.

### Longitudinal fluorescent microscopy and survival analysis

To determine the role of TFEB in cell survival, primary cortical neurons were transfected with pGW1-mApple, as a morphology and viability marker, and pGW1-TFEB-EGFP. To elucidate the effect of HPβCD in cortical primary neurons, primary cortical neurons were transfected with pGW1-mApple, treated with a vehicle or with 50 nM Dox alone or in combination with different concentrations of HPβCD, and then imaged every 24 h for 6 days with the EVOS microscope system. For tracking the same neurons over time, an image of the fiduciary field with neurons on the plate was collected at the first time-point and used as a reference image. Each time the same plate was imaged thereafter, the fiduciary image was aligned with the reference image. Neurons that died during the imaging interval were assigned a survival time. These event times were used to obtain the exponential cumulative survival graphs and analyzed for statistical significance by Log-Rank test with JMP software (SAS Institute) as described [38, 49]. One hundred to 150 neurons were analyzed from three independent experiments per condition.

### Western blotting

Cultured neurons were collected in RIPA lysis buffer [150 mM NaCl, 1% Nonidet P40, 0.5% sodium deoxycholate, 0.1% SDS and 50 mM Tris/HCl (pH 8.0)] with a mix of protease and phosphatase inhibitors. Cellular suspensions were vortexed and centrifuged at 12.000 g for 10 min at 4°C. Supernatants were collected, and protein concentration was determined using the BCA (bicinchoninic acid) Protein Assay Kit (Thermo Scientific), according to the manufacturer's instructions. Samples were analyzed by SDS/PAGE (4–12% and 16% gels, and proteins were transferred onto PVDF membranes by the iBlot2 system (Life Technologies). Membranes were blocked and incubated overnight with anti-tubulin, anti-actin, anti-p62 or anti-LC3. Membranes were washed with TBS [Tris-buffered saline; 10 mM Tris/HCl and 150 mM NaCl (pH 7.4)] and probed for 1 h with anti-rabbit or anti-mouse antibodies conjugated with horseradish peroxidase. Signals were detected using Lumi-Light Western Blotting Substrate (Roche Applied Science, Indianapolis, IN) on Medical X-Ray Film (Kodak, Rochester, NJ).

### Mice

Female nude mice (Taconic Farms, Hudson, NY) were kept under pathogen-free conditions in facilities approved by the American Association for Laboratory Animal Care. Mice used in the experiments were 4–6 months of age. Mice were intraperitoneally injected with Doxil (Janssen Products, Beerse, Belgium; #NDC 59676-0960-01). Mice were injected weekly (4 mg/kg/week in 150 μl of sterile phosphate-buffered saline (PBS)) for 8 weeks. These conditions recapitulate a dose schedule used in human patients [62]. At 24 h after the last injection, two mice were anesthetized with isoflourane. The chest cavity was opened, and 20 ml of PBS were perfused into the left ventricle of the heart to purge the animal of blood. The animals were then perfused with 30 ml of 2.0% EM grade paraformaldehyde + 2.5% EM grade glutaraldehyde + 0.13 M sucrose in 0.1 M cacodylate buffer, pH 7.4.

### Electron microscopy

The frontal cortices were dissected from the fixed mouse brains, minced into 1-mm cubes, and fixed for additional 2 days at 4°C. After primary fixation, the tissues were washed in 0.1 M cacodylate buffer and post-fixed with 1% cacodylate-buffered osmium tetroxide and 0.8% potassium ferricyanide for 90 minutes. The samples were washed several times in water, then dehydrated in increasing concentrations of ethanol, infiltrated, and embedded in Spurr's Low Viscosity embedding medium. The samples were polymerized in a 62°C oven for 3 days. Ultrathin sections were cut at 60 nm on a Leica U7 ultramicrotome (Leica, Deerfield, IL), stained with saturated aqueous uranyl acetate and counterstained with Reynold's lead citrate. The sections were examined in a Hitachi H7500 transmission electron microscope using an accelerating voltage of 80 kV, and images were captured via an AMT XR-16 digital camera and AMT Image Capture, v602.600.51 software.

### Ethics statement

Rats and mice were maintained in accordance with guidelines and regulations of the University of Texas, Houston, and the MD Anderson Cancer Center Institutional Animal Care and Use Committee (the protocol numbers #AWC-13-122, #AWC-16-0081, and #00001029-RN01). All experimental protocols were approved by the University of Texas, Houston, and by MD Anderson Cancer Center. The methods were carried out in accordance with the approved guidelines.
